# Microarray Data Analysis of Gene Expression Evolution

**DOI:** 10.4137/grsb.s2997

**Published:** 2009-11-27

**Authors:** Honghuang Lin

**Affiliations:** Dana-Farber Cancer Institute, Harvard Medical School, Boston, MA 02115, USA. Email: honghuang_lin@dfci.harvard.edu

**Keywords:** microarray, data analysis, gene expression evolution, normalization, function enrichment

## Abstract

Microarrays are becoming a widely used tool to study gene expression evolution. A recent paper by Wang and Rekaya describes a comprehensive study of gene expression evolution by microarray.^1^ The work provides a perspective to study gene expression evolution in terms of functional enrichment and promoter conservation. It was found that gene expression patterns are highly conserved in some biological processes, but the correlation between promoter and gene expression is insignificant. This scope of this work and future improvement to study gene expression evolution will be discussed in this article.

The advance of microarray technology enables scientists to monitor the expression profile of thousands of genes simultaneously, making it a possible tool to study transcriptome evolution. Microarrays have been widely used to study expression relationship between humans and other organisms.^2^–^6^ The rationale behind these studies is that orthologous tissues carry out similar physiological functions, which suggests that they are likely to have similar expression profiles. In particular, the expression profile should be conserved for functionally important genes.

A recent paper by Wang and Rekaya describes a comprehensive study of gene expression evolution between humans and mice.^1^ Two human/mouse gene expression data sets^2^,^7^ and one yeast expression data set^8^ were analyzed. The expression similarity was measured by two methods, relative abundance (RA)^5^ and all one-to-one ortholog pairs.^9^ Significant expression conservation was observed between functional related genes in terms of gene ontology (GO). Such conservation could be found in both related species (human vs. mouse) and distant species (human vs. yeast). The authors proposed that events like gene duplication and speciation might result in conservation loss. Expression conservation is not solely dependent on the degree of sequence identity or evolutionary divergence time.^1^,^9^ Similar results were also observed in previous studies.^4^–^6^ It should be noted that GO is not always be the only or most appropriate source of gene functional annotation. Knowledge from other sources, such as DAVID,^10^ Pfam,^11^ and UniProt,^12^ might be adopted in the future study.

Wang and Rekaya also investigated the correlation between promoter sequences and gene expression based on global alignment, local alignment and motif-count. Weak correlation was observed between humans and mice. Such correlation, however, was not observed between humans and yeast, suggesting different regulatory mechanisms might be involved in these two species.^1^ Moreover, promoter function is highly context dependent, which limits the capability of homology search for functional annotation.^13^ Duplication and transposition of DNA motifs might also result in promoter mutations together with nucleotide mutations.^1^

The expression divergence between species is likely to be overestimated due to various factors. The expression of each gene is usually interrogated by multiple probes called a probeset. The intensity signals from each probe in a probeset are then summarized to obtain the overall expression measurement for the gene.^14^–^16^ Different probesets for the same gene in different species might have different sensitivity, which might result in low correlation of expression profiles for between-species comparison.^5^ It was estimated that the measurement error is likely to be attributable to the majority of expression divergence observed in microarray data.^5^ Liao et al. introduced relative abundance (RA) to measure the relative expression level of a gene in a given tissue among the sampled tissues, which showed better performance than using gene measurement alone.^5^ The method was also adopted in Wang and Rekaya’s study, and succeeded in identifying highly conserved functional groups. Other factors, such as DNA methylation, RNA alternative splicing, and transcription factor co-evolution, could also affect gene expression.^13^,^17^ Cross-hybridization is another cause attributable to the inaccurate signal measurement. Some studies found that excluding suboptimal probesets would reduce the effects of cross-hybridization,^18^ although its significance is still controversial.^5^ Gene expression profiling is usually studied under different experimental conditions, cell types, and development stages, resulting in divergent sets of genes expressed. A subset, such as a pathway, could be studied, instead of the whole sets of unrelated microarray data, to avoid the overall complexity.^4^,^19^

Systematic bias might be introduced during the preparation of sample libraries, hybridization, or image scanning. Proper normalization is thus an essential step in gene expression evolution study. The simplest normalization method is to adjust array signals according to the global signal median, which would, on the other hand, result in local intensity bias. Lowes normalization is a widely used normalization method. It applies a locally weighted linear regression to eliminate intensity-dependent local biases, making it robust to outliers.^20^ Quantile method normalizes the distribution of probe intensities across different arrays to a baseline, usually the sample with median intensities. In practice, quantile normalization is recommended to be used for gene expression evolution due to its low variance and bias.^21^ A flow-chart of typical steps involving in microarray data analysis of gene expression evolution is shown in Figure 1.

Overall, the work by Wang and Rekaya provides a functional significance approach to investigating gene expression evolution between humans and mice. Coupled with technologies to alleviate the negative effects from experimental variation, cross hybridization and systematic bias, microarray would become a powerful tool to study gene expression evolution.

## Figures and Tables

**Figure 1.**
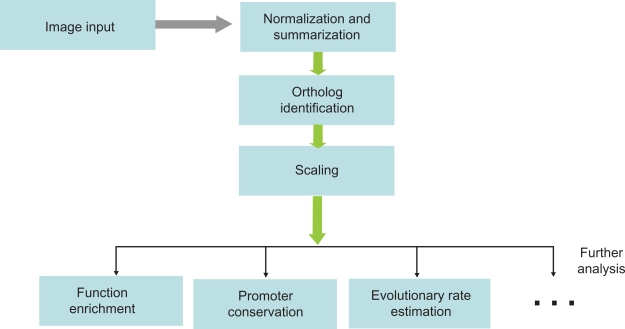
A flowchart of typical steps involving in microarray data analysis of gene expression evolution.
